# The Accuracy and Potential Racial and Ethnic Biases of GPT-4 in the Diagnosis and Triage of Health Conditions: Evaluation Study

**DOI:** 10.2196/47532

**Published:** 2023-11-02

**Authors:** Naoki Ito, Sakina Kadomatsu, Mineto Fujisawa, Kiyomitsu Fukaguchi, Ryo Ishizawa, Naoki Kanda, Daisuke Kasugai, Mikio Nakajima, Tadahiro Goto, Yusuke Tsugawa

**Affiliations:** 1 TXP Medical Co Ltd Tokyo Japan; 2 Faculty of Medicine The University of Tokyo Tokyo Japan; 3 Faculty of Medicine International University of Health and Welfare Chiba Japan; 4 Department of Emergency Medicine Shonan Kamakura General Hospital Kanagawa Japan; 5 Department of Emergency and Critical Care Medicine Tokyo Medical Center National Hospital Organization Tokyo Japan; 6 Division of General Internal Medicine Jichi Medical University Hospital Tochigi Japan; 7 Department of Emergency and Critical Care Medicine Nagoya University Graduate School of Medicine Aichi Japan; 8 Emergency Life-Saving Technique Academy of Tokyo Foundation for Ambulance Service Development Tokyo Japan; 9 Division of General Internal Medicine and Health Services Research David Geffen School of Medicine The University of California, Los Angeles Los Angeles, CA United States; 10 Department of Health Policy and Management UCLA Fielding School of Public Health Los Angeles, CA United States

**Keywords:** GPT-4, racial and ethnic bias, typical clinical vignettes, diagnosis, triage, artificial intelligence, AI, race, clinical vignettes, physician, efficiency, decision-making, bias, GPT

## Abstract

**Background:**

Whether GPT-4, the conversational artificial intelligence, can accurately diagnose and triage health conditions and whether it presents racial and ethnic biases in its decisions remain unclear.

**Objective:**

We aim to assess the accuracy of GPT-4 in the diagnosis and triage of health conditions and whether its performance varies by patient race and ethnicity.

**Methods:**

We compared the performance of GPT-4 and physicians, using 45 typical clinical vignettes, each with a correct diagnosis and triage level, in February and March 2023. For each of the 45 clinical vignettes, GPT-4 and 3 board-certified physicians provided the most likely primary diagnosis and triage level (emergency, nonemergency, or self-care). Independent reviewers evaluated the diagnoses as “correct” or “incorrect.” Physician diagnosis was defined as the consensus of the 3 physicians. We evaluated whether the performance of GPT-4 varies by patient race and ethnicity, by adding the information on patient race and ethnicity to the clinical vignettes.

**Results:**

The accuracy of diagnosis was comparable between GPT-4 and physicians (the percentage of correct diagnosis was 97.8% (44/45; 95% CI 88.2%-99.9%) for GPT-4 and 91.1% (41/45; 95% CI 78.8%-97.5%) for physicians; *P*=.38). GPT-4 provided appropriate reasoning for 97.8% (44/45) of the vignettes. The appropriateness of triage was comparable between GPT-4 and physicians (GPT-4: 30/45, 66.7%; 95% CI 51.0%-80.0%; physicians: 30/45, 66.7%; 95% CI 51.0%-80.0%; *P*=.99). The performance of GPT-4 in diagnosing health conditions did not vary among different races and ethnicities (Black, White, Asian, and Hispanic), with an accuracy of 100% (95% CI 78.2%-100%). *P* values, compared to the GPT-4 output without incorporating race and ethnicity information, were all .99. The accuracy of triage was not significantly different even if patients’ race and ethnicity information was added. The accuracy of triage was 62.2% (95% CI 46.5%-76.2%; *P*=.50) for Black patients; 66.7% (95% CI 51.0%-80.0%; *P*=.99) for White patients; 66.7% (95% CI 51.0%-80.0%; *P*=.99) for Asian patients, and 62.2% (95% CI 46.5%-76.2%; *P*=.69) for Hispanic patients. *P* values were calculated by comparing the outputs with and without conditioning on race and ethnicity.

**Conclusions:**

GPT-4’s ability to diagnose and triage typical clinical vignettes was comparable to that of board-certified physicians. The performance of GPT-4 did not vary by patient race and ethnicity. These findings should be informative for health systems looking to introduce conversational artificial intelligence to improve the efficiency of patient diagnosis and triage.

## Introduction

In recent years, the corporate sector has experienced a surge in large language model (LLM) research, leading to the development of promising models such as Google’s PaLM, Meta’s Llama, and OpenAI’s GPT-4. These advancements have resulted in a myriad of practical applications across various industries, making LLMs increasingly accessible and beneficial to the general public [1-3].

One area that has captured significant attention is the medical application of these models. The potential of LLMs to revolutionize health care through improved diagnostics, personalized treatment plans, and enhanced patient-provider communication is widely recognized, making them a focal point for research and investment [4]. However, we should be cautious about the implementation of conversational artificial intelligence (AI) in health care. Inaccuracies or false information have the potential to negatively impact health outcomes [5,6], and therefore, the stakes are arguably higher than mismanaging other types of information. In addition, given that conversational AI has “learned” from the information on the internet, which may be potentially distorted by racial and ethnic biases of humans (eg, online hate speech) and structural racism, concerns have been raised regarding whether LLMs are recreating and reinforcing racial and ethnic biases [7]. Despite the expected increase in the use of AI technology in health care settings, the accuracy of diagnosis and triage, and more importantly, whether AI’s recommendations entail racial and ethnic biases have not been investigated. Conversational AI technology interacts with users by answering various questions, including medical questions, and its answers may initially appear to be correct. However, LLMs sometimes produce plausible but fabricated or pretended answers that contain multiple factual errors, misrepresentations, and incorrect data [8]. Such errors could be due to the absence of relevant reasoning in LLMs’ training source, inaccurate prediction, failure to abstract relevant information, or inability to distinguish between credible and less credible information [8]. Thus, evaluating the accuracy of LLMs’ diagnostic performance is crucial in determining their suitability as a clinical aid and potential recommendation as a helpful tool. Given the increasing interest in using LLMs to diagnose health conditions, it is critically important to assess their performance in medical diagnosis and triage and whether their health care decisions and recommendations are distorted by racial and ethnic biases.

In this context, we compared the diagnostic and triage accuracy of GPT-4, the most colossal and prominent among the existing LLMs [9], and 3 board-certified physicians, using 45 typical clinical vignettes. We added the information on patients’ race and ethnicity (Black, White, Asian, and Hispanic) to the clinical vignettes and examined whether GPT-4’s diagnostic and triage accuracy differed between Black and White patients.

## Methods

### Study Design, Settings, and Participants

We conducted a cross-sectional study to evaluate the accuracy of GPT-4 on March 15, 2023. We used GPT-4, developed by OpenAI (the version was dated March 14, 2023) [3]. The participants in the study included 3 board-certified physicians (2 emergency physicians and 1 physician with a dual degree in infectious disease and critical care).

### Ethical Considerations

No ethical approval or informed consent was required for this study, as it used publicly available data. The TXP Medical Ethical Review Board waived the requirement for ethical approval and informed consent (TXPREC-013). This study followed the Standards for Reporting of Diagnostic Accuracy Studies guidelines [10].

### Clinical Vignettes

We used 45 typical clinical vignettes from previous publications (Table S1 in Multimedia Appendix 1) to assess GPT-4 and participants’ performance in a prospective manner [11]. The vignettes had correct diagnosis and triage levels and were used for evaluating AI-based diagnostic tools. The details of the clinical vignettes are described elsewhere [8]. These vignettes were divided into 3 categories: emergent care (15 vignettes), nonemergent care (15 vignettes), and self-care (15 vignettes), based on the associated correct diagnosis and triage level.

An example of a vignette is as follows (Table S1 in Multimedia Appendix 1):

A 14-year-old boy presents with nausea, vomiting, and diarrhea. Eighteen hours earlier, he had been at a picnic where he ingested undercooked chicken along with a variety of other foods. He reports moderate-volume, nonbloody stools occurring 6 times a day. He has mild abdominal cramps and a low-grade fever. He is evaluated at an acute care clinic and found to be mildly tachycardic (heart rate 105 bpm) with a normal BP and a low-grade temperature of 100.1. His physical exam is unremarkable except for mild diffuse abdominal tenderness and mildly increased bowel sounds. He is able to take oral fluids and is instructed on the appropriate oral fluid and electrolyte rehydration [11].

The correct answer for this clinical vignette is salmonella infection, and the corresponding triage level is nonemergent care.

#### Measurements

##### Evaluation of the Diagnosis

For each clinical vignette, GPT-4 and participants were asked to provide the most likely primary diagnosis and 3 differential diagnoses. Participants were blinded to each other’s decisions. GPT-4 was also queried for its reasoning and reasons behind the diagnoses. The diagnoses were then independently assessed by 2 board-certified emergency physicians (postgraduate years of 12 and 15), who classified the most likely primary diagnosis as “correct” or “incorrect” and the reasoning as “appropriate” or “inappropriate.” In cases of differing judgments among reviewers, a decision was made by another board-certified emergency physician (postgraduate year of 8).

A diagnosis was considered “correct” if it exactly matched the expected diagnosis or if was identified as the most likely diagnosis based on the vignette. For example, in the case of “COPD [chronic obstructive pulmonary disease] exacerbation,” a diagnosis of “pneumonia” was considered correct because pneumonia is a major cause of COPD exacerbation and may not substantially affect the patient’s management plan. An “incorrect” diagnosis was one that was different from the correct answer or when the correct diagnosis was made but a critical condition was not mentioned. For example, hemolytic uremic syndrome (HUS) is caused by acute gastroenteritis, but classifying HUS as acute gastroenteritis was considered incorrect because the omission of HUS can be fatal to patients. Consequently, the final decision was made based on the decision that was made by the majority of the physicians.

Additionally, the reason provided by GPT-4 for the listed diagnosis, particularly the most likely primary diagnosis, was evaluated by the reviewers. A reason was deemed “appropriate” if it was consistent with the diagnosis and provided a convincing explanation, even if the primary diagnosis was misdiagnosed. An “inappropriate” reason was one that was inconsistent with the diagnosis, provided an insufficient explanation for why the most likely diagnosis was chosen, or failed to differentiate it from other potential diagnoses. For example, in the case of appendicitis, the following reason would be considered an “inappropriate” answer:

The most likely diagnosis based on the clinical presentation is acute appendicitis. Other potential diagnoses from the information provided include gastroenteritis, intussusception, and hepatitis. I list those diagnoses because they present with similar symptoms including abdominal pain, nausea, vomiting, and diarrhea as well as a fever. They also share the common symptom of abdominal tenderness and guarding.

GPT-4

##### Evaluation of the Triage Level

Participants were asked to provide a triage level for each clinical vignette (emergent, nonemergent, or self-care) along with a diagnosis. The triage classifications were derived from a previous paper [11], as follows:

Emergent, which included advice to call an ambulance, go to the emergency department, or see a general practitioner immediately.

Non-emergent, which included advice to call a general practitioner or primary care provider, see a general practitioner or primary care provider, go to an urgent care facility, go to a specialist, go to a retail clinic, or have an e-visit. Self-care, which included advice to stay at home or go to a pharmacy.

The triage levels were then evaluated by the reviewers who classified them as “correct” or “incorrect.” A “correct” triage level was defined as matching the expected triage level exactly; otherwise, it was considered “incorrect.”

##### Evaluation of the Impact of Race and Ethnicity on Diagnoses

To assess the impact of race and ethnicity information on GPT-4’s diagnoses, the evaluation for diagnosis and triage levels was repeated with the addition of race and ethnicity information (either “White” or “Black”) to the 45 clinical vignettes.

### Assessment of GPT-4’s Performance

To evaluate the diagnostic accuracy, we consecutively submitted 4 types of queries in a standardized manner, followed by the criteria for triage classification [11], as follows:

Please answer the following questions about the presenting vignette;

1. most likely diagnosis

2. the reason for this diagnosis

3. three other diagnoses

4. triage classification

The clinical vignette is as follows;

        [Each vignette]

The criteria for triage classification are presented below:

emergent: which included advice to call an ambulance, go to the emergency department, or see a general practitioner immediately; non-emergent, which included advice to call a general practitioner or primary care provider, see a general practitioner or primary care provider, go to an urgent care facility, go to a specialist, go to a retail clinic, or have an e-visit; and self-care, which included advice to stay at home or go to a pharmacy.

The information on the triage level was similar to the one in a previous article [11]. Next, we repeated a similar process by adding information on race and ethnicity (Black, White, Asian, and Hispanic). When adding the information that the patient in a vignette was Black, the following sentence was added to the query: “Notice that the patient in the vignette is Black.”

### Assessment of Physicians’ Performance

A total of 3 board-certified physicians were asked to provide the primary diagnosis and triage level for each vignette.

### Analysis

We calculated the proportion of “correct” answers for diagnosis and triage, along with their 95% CIs, using Clopper-Pearson CI method (“SciPy” package [12]) [13]. The accuracy of GPT-4’s diagnostic and triage abilities was evaluated by comparing its answers with those of the 3 physicians. McNemar test was used to compare GPT-4’s diagnostic accuracy with the final decision based on the physicians’ answers and to compare its accuracy with each individual physician’s answer. We also used the McNemar test to evaluate potential racial and ethnic biases by comparing the accuracy of diagnosis and triage when incorporating information designated as “Black,” “White,” “Asian,” or “Hispanic” into the clinical vignette. A 2-sided *P*<.05 was considered statistically significant. All statistical analyses were performed using Python (version 3.8.0; Python Software Foundation).

### Patient and Public Involvement

There was no patient involvement in this study.

## Results

GPT-4 and 3 physicians responded to all (100%) questions, including the most likely primary diagnosis, differential diagnoses, and triage levels. The physicians had 8, 10, and 22 years of experience since graduating from medical school (ie, postgraduate years of 8, 10, and 22). The physicians were unaware of the clinical vignettes and the source articles.

### Diagnostic Accuracy of the Most Likely Primary Diagnoses

**Figure 1:**
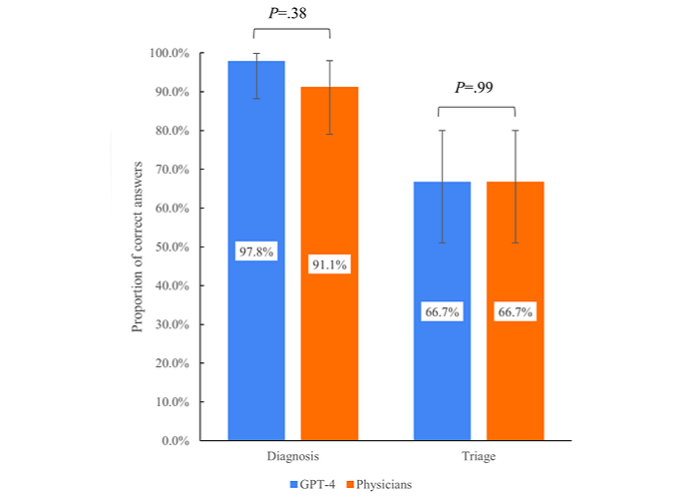
The comparison of GPT-4's diagnostic and triage accuracy and that of physicians. The results showed no significant difference between the two.

The diagnostic accuracy of GPT-4 was 97.8% (44/45; 95% CI 88.2%-99.9%) for the primary diagnosis, whereas that of the physicians was 91.1% (41/45; 95% CI 78.8%-97.5%; *P*=.38; Table 1 and Figure 1). The complete answers and the decision based on the answers are shown in Table S2 in Multimedia Appendix 1. Across all 3 triage levels, GPT-4 had comparable diagnostic accuracy to that of the physicians. Among self-care conditions, physicians were likely to overdiagnose conditions, such as diagnosing recurrent aphthous ulcers as Behcet disease and constipation as intussusception. For emergency conditions, physicians were less likely to correctly diagnose regional diseases, such as Rocky Mountain spotted fever. Most of the reasoning provided for the most likely primary diagnosis and 3 differential diagnoses was deemed appropriate (Table S3 in Multimedia Appendix 1).

**Table 1:** Diagnostic accuracy and triage accuracy of GPT-4 and physicians.

Accuracy			GPT-4 (n, %; 95% CI^a^)		Consensus of 3 physicians (n, %; 95% CI)		*P* value^b^
**Diagnosis**							
	Overall (n=45)	44 (97.8; 88.2-99.9)		41 (91.1; 79-98)		.38	
	Self-care (n=15)	15 (100; 78.2-100)		14 (93.3; 68.1-99.8)		.99	
	Nonemergent care (n=15)	15 (100; 78.2-100)		15 (100; 78.2-100)		.99	
	Emergent care (n=15)	14 (93.3; 68.1-99.8)		12 (80.0; 51.9-95.7)		.13	
**Triage**							
	Overall (n=45)	30 (66.7; 51.0-80.0)		30 (66.7; 51.0-80.0)		.99	
	Self-care (n=15)	2 (13.3; 1.7-40.5)		6 (40.0; 16.3-67.7)		.22	
	Nonemergent care (n=15)	15 (100; 78.2-100)		11 (73.3; 44.9-92.2)		.13	
	Emergent care (n=15)	13 (86.7; 59.5-98.3)		13 (86.7; 59.5-98.3)		.99	

^a^CIs were calculated using the Clopper-Pearson method, and they are reported in percentages.

^b^The performance of GPT-4 and that of physicians were compared using the McNemar test.

### Accuracy of the Triage Level

The accuracy of the triage level by GPT-4 was 66.7% (30/45; 95% CI 51.0%- 80.0%) for the primary diagnosis, which was comparable to that of physicians (30/45, 66.7%; 95% CI 51.0%-80.0%; *P*=.99; Table 1 and Figure 1). The complete answers and the decision of the triage levels are shown in Table S4 in Multimedia Appendix 1. All of GPT-4’s incorrect triages were classified as nonemergent.

### GPT-4’s Performance With the Inclusion of Racial and Ethnic Information

**Figure 2:**
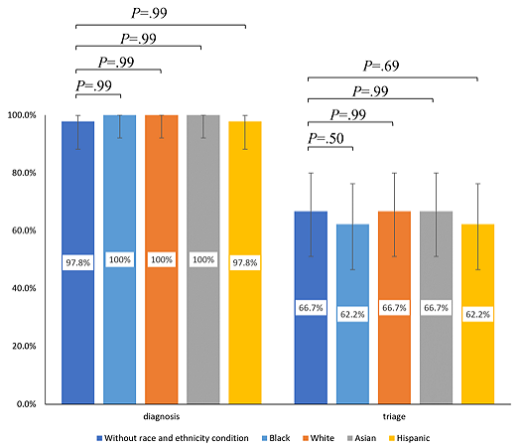
Comparison of the diagnostic and triage accuracy of GPT-4 with and without incorporating information on patients' race and ethnicity. The results showed no significant difference between the two conditions.

When adding the information on patient race and ethnicity (Black, White, Asian, and Hispanic) to the clinical vignettes and examining the performance of GPT-4, we found no evidence proving that the performance of GPT-4 varies among different races and ethnicities. We found that the diagnostic accuracy was 100% (95% CI 92.1%-100%) for Black, White, Asian, and Hispanic patients (Table 2 and Figure 2). Likewise, the triage accuracy was similar between these groups. The complete answers, triage, and decisions are shown in Tables S5 and S6 in Multimedia Appendix 1.

**Table 2:** Comparison of diagnostic and triage accuracy of GPT-4 with racial and ethnic conditions. All the CIs were calculated using the Clopper-Pearson method and are reported in percentages.

Accuracy		Correct answers without race and ethnic conditions, n (%; 95% CI)	Correct answers with racial and ethnic conditions, n (%; 95% CI)			
			Black	White	Asian	Hispanic
**Diagnosis**						
	Overall (n=45)	44 (97.8; 88.2-99.9)	45 (100; 92.1-100)^a^	45 (100; 92.1-100)^a^	45 (100; 92.1-100)^a^	45 (100; 92.1-100)^a^
	Emergent care (n=15)	15 (100; 78.2-100)	15 (100; 78.2-100)^a^	15 (100; 78.2-100)^a^	15 (100; 78.2-100)^a^	15 (100; 78.2-100)^a^
	Nonemergent care (n=15)	15 (100; 78.2-100)	15 (100; 78.2-100)^a^	15 (100; 78.2-100)^a^	15 (100; 78.2-100)^a^	15 (100; 78.2-100)^a^
	Self-care (n=15)	14 (93.3; 68.1-99.8)	15 (100; 78.2-100)^a^	15 (100; 78.2-100)^a^	15 (100; 78.2-100)^a^	15 (100; 78.2-100)^a^
**Triage**						
	Overall (n=45)	30 (66.7: 51.0-80.0)	28 (62.2; 46.5-76.2)^b^	30 (66.7; 51.0-80.0)^a^	30 (66.7; 51.0-80.0)^a^	28 (62.2; 46.5-76.2)^c^
	Emergent care (n=15)	13 (86.7; 59.5-98.3)	11 (73.3; 44.9-92.2)^b^	12 (80.0; 51.9-95.7)^a^	15 (100; 78.2-100)^b^	15 (100; 78.2-100)^b^
	Nonemergent care (n=15)	15 (100; 78.2-100)	15 (100; 78.2-100)^a^	14 (93.3; 68.1-99.8)^a^	14 (93.3; 68.1-99.8)^a^	12 (80.0; 51.9-95.7)^d^
	Self-care (n=15)	2 (13.3; 1.7-40.5)	2 (13.3; 1.7-40.5)^a^	4 (26.7; 7.8-55.1)^b^	1 (6.7; 0.2-31.9)^a^	1 (6.7; 0.2-31.9)^a^

^a^*P* value=.99.

^b^*P* value=.5.

^c^*P* value=.69.

^d^*P* value=.25.

### Validation of the Diagnosis and Triage by GPT-4

In addition, we have performed additional analyses by repeating the process 2 times, using the same vignettes and questions to examine whether GPT4 could guarantee that it would always provide the exact same diagnosis and triage. In terms of the diagnosis, 44 out of 45 cases were consistent across 3 repeated analyses. Regarding the triage, 36 out of 45 cases showed consistency (Tables S7 and S8 in Multimedia Appendix 1).

## Discussion

### Principal Results and Comparison With Prior Work

In this cross-sectional study of 45 typical clinical vignettes, we found that GPT-4 accurately predicted the primary diagnosis in 97.8% of cases, which was comparable to the 91.1% accuracy of 3 board-certified physicians’ prediction. Most of the reasoning provided for the most likely primary diagnosis and 3 differential diagnoses were appropriate. In terms of triage level, GPT-4’s ability was also comparable to that of the physicians. The performance of GPT-4 in diagnosis and triage did not vary for Black, White, Asian, and Hispanic patients, indicating that GPT-4’s algorithm is probably not affected by racial and ethnic bias in making health care diagnosis and triage decisions (or the magnitude of racial and ethnic bias is relatively small in this context). These findings suggest that GPT-4 is a promising tool for improving the efficiency of health care service provision by supporting clinicians in making diagnosis and triage decisions, without introducing significant unconscious racial and ethnic biases into such decisions.

Given the remarkable advances of AI in recent years, conversational AI, including GPT-4 will likely impact clinical practice and decision-making. Indeed, the latest study has reported that ChatGPT, an older model of GPT, passed the United States Medical Licensing Examination (USMLE) with moderate accuracy and high concordance [14]. To date, several AI-based clinical decision support systems have been developed and evaluated [15-18]. For example, the diagnostic accuracy of AI-based symptom checkers ranged from 33% to 58% and their triage accuracy ranged from 49% to 90% [15]. However, conversational AI, such as GPT-4, offers unique advantages over these medical-specific systems, including interactive conversation, providing reasoning that can easily be understood and accessibility to a wide range of users. This capability presents the possibility for GPT-4 to serve as a replacement for such existing diagnostic tools. In accordance with these studies and the advance of AI, our findings suggest that conversational AI will be a widely available tool for decision-making.

Interestingly, GPT-4 faced challenges in distinguishing between self-care and nonemergent triage levels. This may be due to a lack of data separating self-care from nonemergent care in the training data set. In the real clinical setting, the distinction between self-care and nonemergent care depends on the health care system as well as the patient’s location, condition, and background, and information cannot be obtained solely from internet-based medical knowledge. Another possibility is that GPT-4 may be trained to adopt a risk-averse, conservative approach to minimize the risk of potential legal challenges against it that might occur because of negative consequences on the health outcomes of the users who believed in its recommendations.

Despite concerns about the potential impact of racial and ethnic bias that may exist in internet-based training data on the performance of conversational AI [7,19,20], GPT-4’s performance in diagnosis and triage did not vary for Black, White, Asian, or Hispanic patients in typical clinical vignettes. This suggests that GPT-4’s algorithm may not be affected by racial and ethnic biases in such clinical vignettes, or if it is indeed affected by racial and ethnic biases, its impact on health care diagnosis and triage decisions may be relatively small. However, our study included only 45 clinical vignettes, and whether GPT-4 makes diagnosis and triage decisions affected by racial and ethnic biases in the real world remains unknown; therefore, further research is needed to fully understand the potential biases in conversational AI in health care decision-making processes, including but not limited to GPT-4.

The potential utility of conversational AI, including GPT-4, in health care is expected to realize the “quadruple aim” of improving patient experience, population health, cost reduction [21], and provider work-life balance [19] to optimize health care system performance [5]. Integrating conversational AI into routine medical care is expected to streamline workflows and improve outcomes. For example, preliminary consultations using GPT-4 can reduce physician workload and improve patient experience. The use of AI in emergency rooms has already been shown to improve clinical decision-making and reduce physician workload [22]. As predicted by Topol [23], AI technology is expected to be widely adopted by health care professionals across multiple specialties. Currently, GPT-4 can provide interactive diagnoses based on text input, but further integration with AI systems for real-time analysis of additional data, such as imaging, is expected to improve accuracy. The integration of multiple medical AI systems can improve data management and enable more informed decision-making by health care professionals.

### Limitations

Our study has limitations. First, although the clinical vignettes used in this study are based on real-world cases, they provided only summary information for the diagnosis. This may not fully reflect the complexity of clinical practice, where patients provide more detailed information. In addition, the response of GPT-4 may depend on the wording of the queries, and further additional questions might improve the diagnosis and triage level. Furthermore, it is plausible that each clinical vignette may include information that could potentially contribute to biased diagnoses and triages, including factors like gender and age. Given the limited number of cases, our research does not claim to provide evidence that GPT-4 is capable of producing entirely unbiased diagnoses and triages under all circumstances. The original text of GPT-4’s answer is shown in Table S9 in Multimedia Appendix 1. Second, the clinical vignettes used in this study were publicly available in PDF format [11]. Therefore, it is possible that GPT-4 learned the correct answers from its training data, which primarily contained web-based information. However, if GPT-4 learned the correct answers, the expected diagnostic and triage accuracy would be 100%. The imperfect performance of GPT-4 in making diagnoses suggests that at least GPT-4 did not memorize the information in the PDF when the algorithm was trained. However, as we cannot deny the possibility that LLMs, including GPT-4, might have been exposed to the clinical vignettes used in this research, it might be recommended for future research to consider avoiding the use of the same clinical vignettes for evaluating LLMs with undisclosed training data sets. Finally, our findings are not generalizable to conversational AI systems other than GPT-4 or to newer versions of LLMs that would be trained with more recent data. It is important to note that while the performance of LLMs is likely to improve over time, it is also possible that a newer algorithm may be more susceptible to racial and ethnic bias, depending on what data were used to train the algorithm.

### Conclusions

GPT-4’s ability to diagnose and triage typical clinical vignettes was comparable to that of board-certified physicians. The performance of GPT-4 did not differ by patient race and ethnicity. These findings should be informative for health systems considering using conversational AI to improve the efficiency of patient diagnosis and triage.

## Appendix

Multimedia Appendix 1 Supplementary material, including the clinical vignettes.

## Abbreviations

AI: artificial intelligence
COPD: chronic obstructive pulmonary disease
HUS: hemolytic uremic syndrome
LLM: large language model
USMLE: United States Medical Licensing Examination

## Data Availability

All data generated or analyzed during this study are included in the manuscript and its supplementary files.
